# Controlled delivery of a protein tyrosine phosphatase inhibitor, SHP099, using cyclodextrin-mediated host–guest interactions in polyelectrolyte multilayer films for cancer therapy†

**DOI:** 10.1039/d0ra03864d

**Published:** 2020-05-27

**Authors:** Soobin Wang, Alessia Battigelli, Dahlia Alkekhia, Alexis Fairman, Valentin Antoci, Wentian Yang, Douglas Moore, Anita Shukla

**Affiliations:** School of Engineering, Brown University Providence RI 02912 USA anita_shukla@brown.edu; Center for Biomedical Engineering, Brown University Providence RI 02912 USA; Department of Orthopaedics, Warren Alpert Medical School, Rhode Island Hospital, Brown University Providence RI 02912 USA; Institute for Molecular and Nanoscale Innovation, Brown University Providence RI 02912 USA

## Abstract

The Src homology 2 domain containing protein tyrosine phosphatase-2 (SHP2) is a key enzyme in pathways regulating tumor growth signaling, and recently gained interest as a promising anticancer drug target. Many SHP2 inhibitors are currently under development, including SHP099, which has shown potent anticancer activity at low concentrations *in vivo*. In this work, we developed multilayer coatings for localized delivery of SHP099 to improve upon current cancer therapies. Layer-by-layer self-assembly was used to develop films composed of chitosan and poly-carboxymethyl-β-cyclodextrin (PβCD) for the delivery of SHP099. SHP099 was successfully loaded into multilayer films *via* host–guest interactions with PβCD. Nuclear magnetic resonance spectroscopy confirmed the occurrence of this supramolecular assembly by identifying the interaction of specific terminal SHP099 protons with the protons of the CD. SHP099 release from assembled films was detected over 96 hours, and was found to inhibit colony formation of human breast adenocarcinoma cells *in vitro*. These multilayer films have the potential to be used in a range of anticancer applications and overcome common complications of systemic chemotherapeutic administration, while maximizing SHP099 efficacy.

## Abstract 2

Controlled delivery of a potent anticancer drug, SHP099, after supramolecular complexation into a versatile multilayer film.

## Graphical abstract

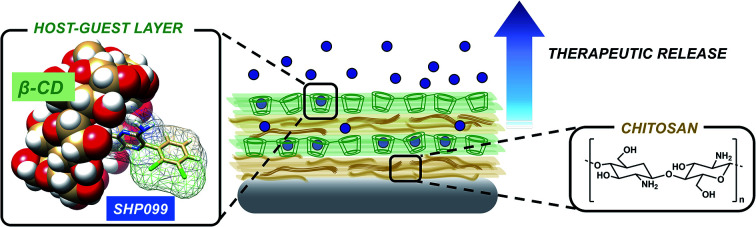

## 1 Introduction

Src homology 2 domain containing protein tyrosine phosphatase-2 (SHP2) is a non-receptor protein tyrosine phosphatase that is ubiquitously expressed and plays a key role in many cellular pathways, regulating development, metabolism, and cell signaling across numerous species.^1,2^ The upregulation of SHP2 has been observed in various cancers (including leukemia, lung and breast cancers, and neuroblastomas) and other malignancies (such as Noonan syndrome and metachondromatosis),^1,2^ leading to interest in targeting SHP2 as a new anticancer drug target. SHP2 has recently been validated as a viable target for cancer therapy,^3^ and there is an increasing focus on identifying SHP2 inhibitors as potential chemotherapeutics.^1,4,5^ From these investigations, SHP099 has emerged as a promising drug candidate.^6–8^ An allosteric inhibitor of SHP2, SHP099 is a recently identified synthetic small molecule which has demonstrated reduced off-target cytotoxicity and high-target specificity against receptor-tyrosine-kinase-driven cancers in *in vitro* and *in vivo* models.^1^ Here we formulated a local delivery system for this promising chemotherapeutic.

As with most chemotherapy, systemically administered drugs circulate until they reach a tumor site where they inhibit intracellular growth pathways, blocking the progression of cancer.^9^ However, the lack of specific tumor targeting causes unintended toxic accumulation in healthy tissue, resulting in adverse side effects^10,11^ and reduced therapeutic efficacy against the tumor.^9^ Thus far, *in vivo* investigations of SHP2 inhibitors have also been plagued by these issues due to difficulties achieving target enzyme specificity and bioavailability without affecting other key enzymatic pathways.^12,13^ Therefore, there is a need for controlled release of these inhibitors and other anticancer drugs through localized or targeted delivery systems.^10^ Polymeric drug delivery systems are of interest for the delivery of chemotherapeutic agents, due to their tunable formulations and ability to deliver small molecules to target sites at therapeutically relevant concentrations.^9^ Of note, layer-by-layer (LbL) self-assembly has gained significant interest for the design of drug delivery systems for cancer therapy, particularly in the coating of nanoparticles for tumor targeting.^14^ LbL assembly is a multilayer film fabrication approach driven by multivalent interactions between complementary species.^15^ Alternating adsorption of these functional molecules (including polyelectrolytes, nucleic acids, proteins, and small molecules, among others) can be used to assemble nanoscale films on a range of substrates.^14,16^ LbL assembly has successfully been used to target cancer cells (*e.g.*, through the selective binding of CD44 cell surface receptors^17,18^); as stimuli-responsive drug delivery systems^19,20^ (*e.g.*, for the release of doxorubicin^21^); or as theranostic agents for magnetic resonance imaging (*e.g.*, after incorporation of gadolinium iii in the film^22^). Despite the tremendous promise of LbL assembly for cancer therapy, this approach has not yet been utilized to deliver SHP2 inhibitors.

**Fig. 1:**
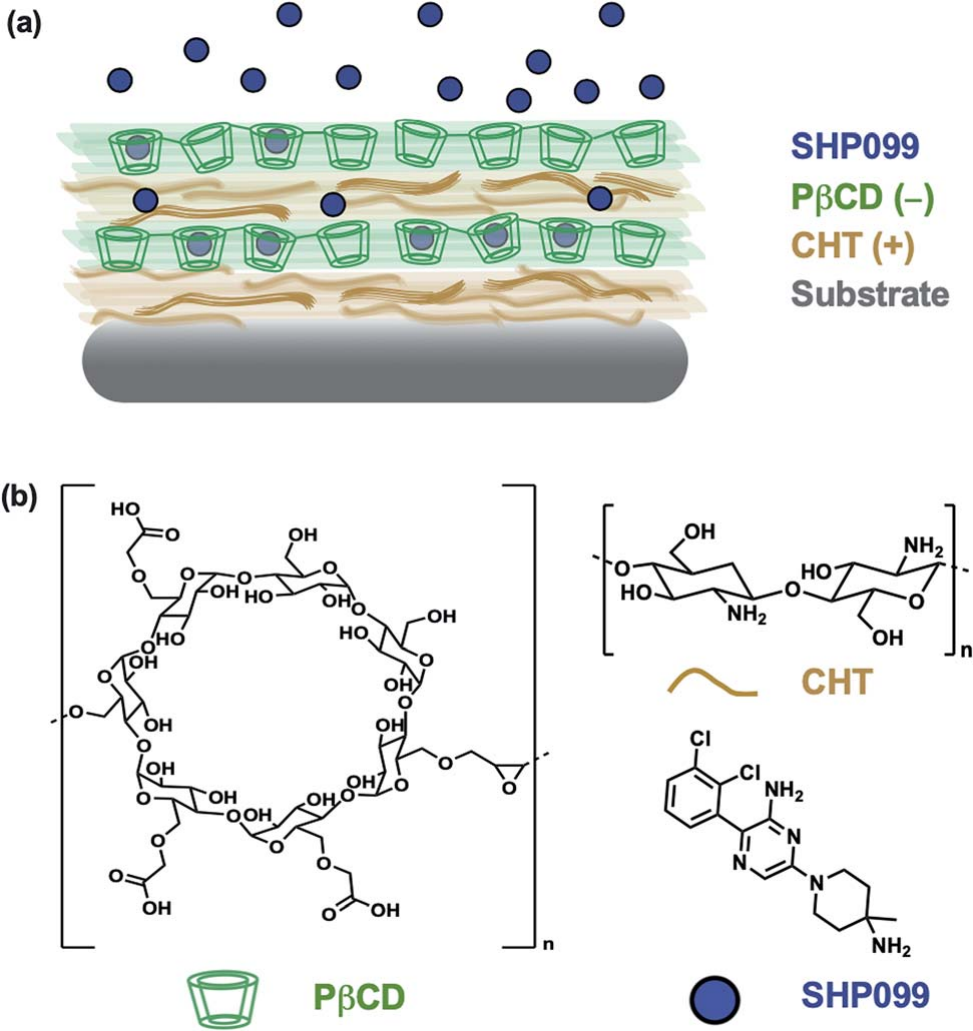
Layer-by-layer self assembly of a SHP099 containing multilayer film. (a) Schematic of (CHT/PβCD–SHP099)_25_ LbL film. (b) Chemical structures of SHP099, poly-carboxymethyl-β-cyclodextrin (PβCD), and chitosan (CHT).

In this study, we investigated the formulation of LbL films for the loading and release of SHP099. We first studied interactions of the SHP099 molecule with β-cyclodextrins (CDs) as a means of loading the hydrophobic small molecule into LbL film architectures *via* host–guest interactions. CDs were chosen as carriers for their known ability to encapsulate hydrophobic small molecules in their hydrophobic core pocket.^23^ Polymerized formulations of CDs or CD-modified polymers have previously been utilized in LbL films for the incorporation and delivery of non-steroidal anti-inflammatory drugs (NSAIDs),^24^ in the development of drug-eluting platforms targeting infections,^25,26^ or to construct nanocarriers for gene delivery.^27^ Here, we investigated poly-carboxymethyl-β-cyclodextrin (PβCD) complexation with SHP099. We developed LbL films by alternating adsorption of chitosan (CHT), a polycation, with the polyanionic, poly-carboxymethyl-β-cyclodextrin complexed with SHP099 (PβCD–SHP099) (Fig. 1). We investigated film assembly properties and SHP099 loading and release, and found that released SHP099 successfully inhibited the proliferation of human breast adenocarcinoma cells *in vitro*.

## 2 Materials and methods

### 2.1 Materials

Chitosan (CHT, ≥75% deacetylated), poly(sodium-4-styrenesulfonate) (SPS, average molecular weight (MW) ∼70 000 Da), methanol, sodium acetate buffer, 10× Dulbecco's phosphate buffered saline (10× PBS, pH 7.4), Dulbecco's Modified Eagle's Medium (DMEM) (containing 4 mM l-glutamine, 4500 mg L^−1^ glucose, 1 mM sodium pyruvate, and 1500 mg L^−1^ sodium bicarbonate), calf bovine serum, and TWEEN® 20 were purchased from Sigma-Aldrich (St. Louis, MO). Linear poly(ethyleneimine) (LPEI, MW ∼45 000 Da) was obtained from Polysciences, Inc. (Warrington, PA), and sodium hydroxide (NaOH) was obtained from Thermo Fisher Scientific (Waltham, MA). Poly-carboxymethyl-β-cyclodextrin (PβCD, MW ∼153 000 Da) was purchased from Cyclo Lab (Budapest, Hungary), while SHP099 dihydrochloride was purchased from ChemieTek (Indianapolis, IN). Deuterium oxide (D_2_O) was purchased from Cambridge Isotope Laboratories (Tewksbury, MA). Silicon wafers were purchased from WaferPro (Santa Clara, CA). MDA-MB-468 human breast adenocarcinoma cells and NIH 3T3 murine fibroblasts were obtained from American Type Culture Collection (ATCC, Manassas, VA). Roswell Park Memorial Institute (RPMI) 1640 medium containing l-glutamine, 4-(2-hydroxyethyl)piperazine-1-ethanesulfonic acid (HEPES), phenol red, sodium pyruvate, high glucose, and low sodium bicarbonate was purchased from Thermo Fisher Scientific (Waltham, MA). Fetal bovine serum (FBS) was obtained from Corning Inc. (Corning, NY) and penicillin–streptomycin was obtained from Caisson Laboratories (Smithfield, UT). Cell counting kit-8 (CCK8) was purchased from Dojindo Molecular Technologies, Inc. (Rockville, MD). Paraformaldehyde (16% w/v) was purchased from Electron Microscopy Sciences (Hatfield, PA). Crystal violet was purchased from Millipore Sigma (St. Louis, MO). Glacial acetic acid was purchased from Fisher Scientific (Hampton, NH). Ultrapure water (18.2 MΩ cm MilliQ water, EMD Millipore, Taunton, MA) was used in all experiments. Room temperature (RT) refers to 21–23 °C.

### 2.2 Characterizing PβCD–SHP099 host–guest complexation using nuclear magnetic resonance

Solutions of 3.08 mg mL^−1^ SHP099, 20.08 mg mL^−1^ PβCD, and SHP099 (3.08 mg mL^−1^) mixed with PβCD (20.08 mg mL^−1^) (corresponding to a 1 : 2 molar ratio relative to the molar mass of PβCD repeat units) were prepared in D_2_O for all nuclear magnetic resonance (NMR) studies. SHP099 and PβCD were mixed in D_2_O at ∼700 rpm for 1.5 hours at RT to allow formation of PβCD complexed with SHP099 (PβCD–SHP099). One-dimensional (1D) proton (^1^H) NMR spectra were acquired using a Bruker DRX Avance 400 MHz spectrometer. Two-dimensional (2D) nuclear Overhauser effect spectroscopy (NOESY) and diffusion ordered spectroscopy (DOSY) experiments were performed using a Bruker Ascend 600 MHz spectrometer.

### 2.3 (CHT/PβCD–SHP099) LbL film assembly and characterization

#### 2.3.1 LbL film assembly

Silicon substrates (∼0.7 × 2.5 cm) were prepared for LbL assembly by first rinsing three times in methanol and three times in ultrapure water, and finally drying with a stream of air following our previous work.^28^ Dried substrates were plasma etched using air in a Harrick PDC-32G plasma cleaner operated at a high radio frequency level (12 MHz) for 1 min. Substrates were immediately submerged in LPEI (10 mM, pH 4.25). All substrates were initially coated with (LPEI/SPS)_10_ films to facilitate subsequent (CHT/PβCD)_*n*_ assembly, where *n* represents the number of adsorbed film bilayers. For (LPEI/SPS)_10_, each film bilayer was adsorbed by submerging the substrates in LPEI (10 mM, pH 4.25) for 5 min, followed by three rinses in ultrapure water for 10, 20, and 30 s, and then SPS (10 mM, pH 4.75) for 5 min, followed by three additional ultrapure water rinses (10, 20, and 30 s).

(CHT/PβCD)_*n*_ films were assembled using a Biolin Scientific KSV Nima dip coater. Substrates were first submerged in CHT (1 mg mL^−1^ in 0.1 M sodium acetate buffer, pH 6) for 10 min, followed by a 1 min rinse in sodium acetate buffer (0.1 M, pH 6) with gentle agitation. These substrates were then submerged in PβCD (20.08 mg mL^−1^ in 0.1 M sodium acetate buffer, pH 6), followed by a 1 min rinse in sodium acetate buffer (0.1 M, pH 6) with gentle agitation. This process was repeated *n* times to assemble the desired number of bilayers.

For assembly of (CHT/PβCD–SHP099)_*n*_, LbL films were either loaded with SHP099 “pre-assembly” or “post-assembly”. For “pre-assembly” loaded films, PβCD–SHP099 was prepared by mixing SHP099 (3.08 mg mL^−1^) and PβCD (20.08 mg mL^−1^) in sodium acetate buffer (0.1 M, pH 6) at ∼700 rpm for 1.5 hours at RT, and using the PβCD–SHP099 in place of PβCD during film assembly. For SHP099 loading “post-assembly” (CHT/PβCD)_25_ films were incubated in 2 mL of SHP099 (3.08 mg mL^−1^ in sodium acetate buffer, pH 6) at RT for 24 hours, shaking at 50 rpm. All LbL films were stored dry at 4 °C for use in subsequent experiments. Any film or complex formed on the non-plasma-treated surface of the substrate was removed using 1 mM NaOH before any further experiments were performed.

#### 2.3.2 Characterization of film growth

Growth of (CHT/PβCD)_*n*_ and (CHT/PβCD–SHP099)_*n*_ “pre-assembly” loaded films was examined by measuring dry film thickness for *n* = 5, 10, 15, 20, and 25 bilayers. A Veeco Dektak 3 surface profilometer was used to measure average roughness and thickness of all films above 200 nm thickness, while a J.A. Woollam M-2000 ellipsometer was used to measure thicknesses of films below 200 nm. For profilometry measurements, films were scratched at 3 different locations along the length of each substrate and the average step height between the uncoated silicon surface and the film was determined at each scratch. Scan lengths of 2000 μm were utilized. Ellipsometry measurements were taken using a 632.8 nm laser at three incidence angles: 55°, 65°, and 75°. The refractive index for the films was set to 1.55. Measurements were taken at 10 different locations on the substrate and averaged.

#### 2.3.3 Quantification of SHP099 loading

To quantify SHP099 loading in (CHT/PβCD–SHP099)_25_, films were first disrupted in 50 μL of 1 M NaOH for 15 min at RT. This NaOH solution, now containing all dissolved film components, was diluted in 950 μL of 1× PBS (pH 7.4). The absorbance was measured at 350 nm (the determined wavelength of maximum absorbance for SHP099) using a BioTek® Cytation 3 plate reader. These values were compared to SHP099 standard curves to quantify the amount of SHP099 loaded in the films.

### 2.4 SHP099 release and change in film thickness over time

SHP099 release profiles from (CHT/PβCD–SHP099)_25_ films were determined by incubating coated substrates in 500 μL of 1× PBS, pH 7.4 at 37 °C. Solutions were refreshed with 1× PBS at hourly time points (over 5 hours) followed by daily time points until release was no longer detected. SHP099 was quantified in each of these solutions by measuring the absorbance at 350 nm using a BioTek® Cytation 3 plate reader and comparing to SHP099 standards. To determine film thickness at each time point, (CHT/PβCD–SHP099)_25_ films were removed from their 1× PBS incubation solutions, dried with a gentle stream of nitrogen, and measured for thickness using a profilometer or ellipsometer (as described in section Characterization of film growth). Following each measurement, the incubation solutions were replaced with fresh 1× PBS.

### 2.5 Clonogenic assays of *in vitro* cell proliferation

To study the effects of SHP099 released over time from (CHT/PβCD–SHP099)_25_ films on cancer cell growth, the film coated silicon wafers were first sterilized *via* exposure to ultraviolet light in a Nuaire Class II Type A2 biosafety cabinet for 15 min per side. These film coated substrates were then incubated in 0.5 mL of cell culture medium (RPMI 1640 with 10% v/v FBS and 1% v/v penicillin/streptomycin) at 37 °C. At each time point (hourly for the first 5 hours, followed by daily time points over 96 hours), the medium was removed and frozen at −20 °C for future testing with cells, and fresh medium was added to the substrates.

MDA-MB-468 human breast adenocarcinoma cells were cultured in RPMI 1640 medium with 10% v/v FBS and 1% v/v penicillin/streptomycin at 37 °C and 5% CO_2_. Cells were seeded in 96-well plates at a density of ∼800 cells per cm^2^; 24 hours post-seeding, cells were treated with: SHP099 (dilutions from 50 to 1.25 μM), SHP099 mixed with PβCD (50 to 1.25 μM of SHP099 mixed with PβCD at a 1 : 2 molar ratio for 1 hour at 37 °C to allow for complexation), PβCD (dilutions from 0.89 to 0.0089 μM), or the previously collected LbL film-incubated culture medium. SHP099, PβCD–SHP099, and PβCD solutions were prepared from stock solutions in 1× PBS and then diluted in MDA-MB-468 media to reach a final concentration of 10% v/v PBS before incubation with cells. Media was replaced every 3–4 days with fresh test solutions. Cells were grown in a 10% PBS in growth media or in growth media only as positive controls.

After 14 days of treatment, cells were washed with 0.01% PBS-TWEEN three times, fixed with 4% w/v paraformaldehyde for 15 min at RT, washed again with PBS-TWEEN three times, and then stained with 0.2% w/v crystal violet in water while shaking at RT for 30 min. Cells were then thoroughly washed with water to remove excess crystal violet and imaged for colony formation using a digital camera. The crystal violet stain was eluted in 33% v/v acetic acid with rocking for 5 min at RT, and the absorbance was measured at 595 nm to quantify cell proliferation. Acetic acid alone was used as a negative control and results are shown after background subtraction.

### 2.6 Statistical analysis

Results are reported as mean ± standard deviation whenever appropriate. All experiments were repeated with three or more samples. Statistical significance was calculated using Student's *t*-test or one-way analysis of variance (ANOVA; *α* = 0.05) with Tukey's post hoc analysis on GraphPad PRISM™. A value of *p* < 0.05 was considered statistically significant (*p < 0.05; **p < 0.01; ***p < 0.001; ****p < 0.0001).

## 3 Results and discussion

### 3.1 Characterizing PβCD–SHP099 host–guest complexation

**Fig. 2:**
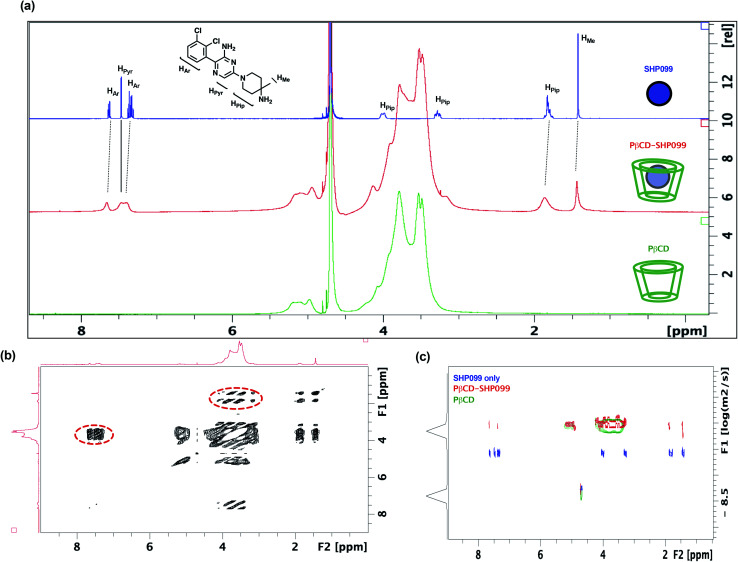
Investigating SHP099 interaction with PβCD using NMR. (a) Comparison of 1D ^1^H-NMR spectra for solutions of SHP099 (blue; 3.08 mg mL^−1^), PβCD–SHP099 (red; 3.08 mg mL^−1^ SHP099, 20.08 mg mL^−1^ PβCD), and PβCD (green; 20.08 mg mL^−1^) dissolved in D_2_O. (b) 2D NOESY spectra of PβCD–SHP099. Through-space coupling between the resonances of SHP099 and PβCD are indicated with red dashed outlines. (c) DOSY spectra of SHP099 (blue), PβCD–SHP099 (red), or PβCD (green).

In this work, we explored the use of PβCD for the loading of the SHP2 inhibitor, SHP099, into LbL films. Given the hydrophobicity of SHP099 (predicted octanol–water partition coefficient log *P*, milog *P* of 3.24, http://www.molinspiration.com), we hypothesized that this molecule would be able to partially enter the hydrophobic pocket of the PβCD repeat unit, which is known to encapsulate hydrophobic molecules smaller than 0.78 nm in diameter, and form host–guest interactions.^23^ In order to understand the nature of potential interactions occurring between SHP099 and PβCD, a combination of 1D and 2D ^1^H-NMR techniques was used (Fig. 2). 1D ^1^H-NMR studies were conducted on SHP099, PβCD, and PβCD–SHP099 (Fig. 2a). Isolated ^1^H-NMR spectra of SHP099 clearly display a set of aromatic and heteroaromatic resonances in the range of 8.00 to 7.00 ppm and the remaining piperidinyl and methyl resonances at 4.00–1.70 and 1.40 ppm, respectively. On the other hand, the NMR spectrum of PβCD shows a broad set of resonances concentrated in the area between 6.00–5.00 and 4.50–3.00 ppm. Such broad resonances are direct consequences of the polymeric nature of PβCD, and have previously been observed for PβCD.^29,30^ Interestingly, when mixed in a 1 : 2 molar ratio of SHP099 to PβCD, we observed a broadening of the aromatic and heteroaromatic SHP099 signals in 1D ^1^H-NMR (Fig. 2a). These differences are attributed to the changing molecular environment of the SHP099, which upon complexation, becomes an integral part of the polymeric PβCD structure causing increases in its nuclei relaxation times. Similar responses have previously been observed in ^1^H-NMR spectra of other CD complexes.^29–31^ Minor differences in chemical shifts of the proton resonance of SHP009 were also observed for PβCD–SHP099 (Fig. 2a). Specifically, downfield shifts of 0.05 and 0.04 ppm were observed for the multiplets of the aromatic protons of SHP099 (Fig. 2a). Additional downfield shifts of 0.02 and 0.06 were also observed in signals corresponding to the methyl and the piperidinyl protons of SHP099, respectively at 1.42 and 1.95 ppm (Fig. 2a). These changes in chemical shifts are consistent with the formation of cyclodextrin inclusion complexes previously reported.^31,32^ Resonances of the remaining two protons of the piperidinyl group (at 3.28 and 4.00 ppm, respectively) were masked by the signal from the PβCD, while no shifts were observed for the proton of the pyrazine group of SHP099 (at 7.47 ppm) (Fig. 2a). The observed resonance shifts of the aromatic and piperidinyl protons, but not of the pyrazine ring proton, suggest drug interaction with PβCD on either or both ends of the drug molecule (*i.e.*, on the dichlorophenyl or piperidinyl groups).

To better understand the structure of the PβCD–SHP099 host–guest complex, 2D NOESY was performed on the PβCD–SHP099 complex mixture (Fig. 2b). These experiments further provided evidence for intermolecular interactions between the cyclodextrin cavity and SHP099. A direct coupling was detected between the piperidinyl fragment of SHP099 and the cyclodextrin resonances of the PβCD, indicating close proximity (<5 Å) between these two groups. A similar coupling was also observed between PβCD and the aromatic portion of SHP099, further highlighting their spatial proximity (Fig. 2b, areas highlighted with dotted line circles). Spectra of SHP099 and PβCD used as controls in these experiments are included in ESI Fig. S1.†

Additionally, 2D-DOSY, a technique that is conventionally used to analyze mixtures of molecules in solution,^33^ was performed to further investigate PβCD–SHP099 interactions. This technique provides important information about the diffusion coefficients of the species present and therefore the size of molecular species formed upon host–guest complexation.^33^Fig. 2c shows a representative 2D-DOSY spectra in which the diffusion coefficients of SHP099, PβCD, and PβCD–SHP099 are shown against their NMR spectra. As expected, SHP099 alone has a greater diffusion coefficient (3.5 × 10^−10^ m^2^ s^−1^) than the diffusion coefficient of the PβCD alone (7.9 × 10^−11^ m^2^ s^−1^), given its significantly smaller molecular size. However, in the mixture of PβCD–SHP099, the SHP099 resonances appeared with diffusion coefficient comparable to the PβCD alone spectra (8.8 × 10^−11^ m^2^ s^−1^); these observations are especially clear for the piperidinyl and the aromatic portion of SHP099. The decrease in diffusion rates further confirms the formation of host–guest interactions deduced from chemical shifts observed in 1D ^1^H-NMR and NOESY NMR experiments. Moreover, the absence of any resonances in the mixture of PβCD–SHP099 exhibiting the same diffusion coefficient as that of SHP099 alone suggests a complete complexation of the drug with the PβCD.

### 3.2 Characterization of SHP099 loading and release from (CHT/PβCD–SHP099)_25_ films

**Fig. 3:**
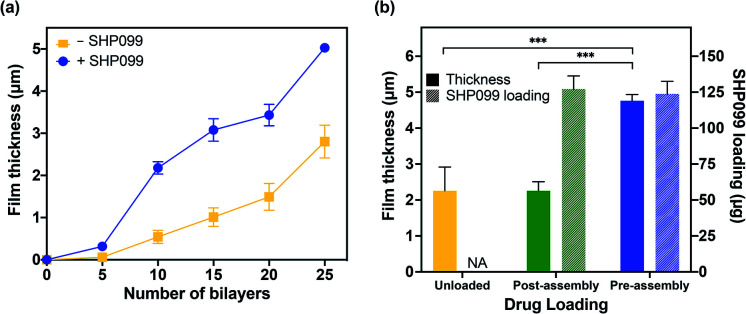
Characterization of LbL film growth and SHP099 loading. (a) Average film thickness of (CHT/PβCD)_*n*_ films, with (+) and without (−) SHP099, with increasing number of bilayers, *n*. (b) Average film thickness and SHP099 loading of “pre-” and “post-assembly” loaded (CHT/PβCD)_25_ films, and unloaded films. Results are reported as mean ± standard deviation; statistical significance was examined using one-way ANOVA and Tukey's post hoc analysis, *n* = 3, *α* = 0.05, ***p < 0.001. NA: not applicable.

Having successfully confirmed the formation of PβCD–SHP099 host–guest interactions, we assembled LbL films based on electrostatic interactions between CHT and the PβCD, both with and without PβCD–SHP099 pre-mixed. CHT is a natural polymer derived from the exoskeleton of shellfish,^34^ and is commonly used in LbL applications due to its biocompatible, biodegradable, and antimicrobial properties.^35,36^ Successful film growth was observed for both (CHT/PβCD)_*n*_ and (CHT/PβCD–SHP099)_*n*_ film architectures (Fig. 3a). However, we observed that these films displayed differing roughness values (1.00 ± 0.05 μm and 0.74 ± 0.16 μm, for 25-bilayer films with and without SHP099, respectively). (CHT/PβCD)_*n*_ and (CHT/PβCD–SHP099)_*n*_ films also exhibited differing growth kinetics. A slow growth regime was observed below 5 bilayers for both films as seen in many LbL films, attributed to the initial formation of islets, *i.e.*, small “islands” of deposited film, as opposed to uniform layers.^37–39^ As additional layers are deposited, these islets grow and coalesce until they form a uniform film surface, facilitating more rapid subsequent film growth. Films containing SHP099 grew more rapidly from 5 to 20 bilayers (∼200 nm per bilayer) compared with the slower growth of films without SHP099 (∼100 nm per bilayer). At 20 bilayers, both film types transitioned to a second faster linear growth regime (∼320 nm per bilayer and ∼260 nm per bilayer for the last 5 bilayers assembled with and without SHP099, respectively). This transition to a more rapid growth has been observed in other LbL film architectures where significant interdiffusion of layers has been observed.^28,40,41^

We compared loading of SHP099 in LbL films formed *via* (1) “pre-assembly” mixing of SHP099 with PβCD prior to film assembly and (2) “post-assembly” SHP099 loading by incubating (CHT/PβCD)_25_ films in SHP099 solution following film assembly. Both drug loading and release profiles of these films were examined. “Pre-assembly” loaded LbL films were approximately twice as thick and rough as “post-assembly” loaded films (thicknesses of 5.03 ± 0.11 μm *versus* 2.26 ± 0.25 μm, and average roughness values of 1.00 ± 0.05 μm *versus* 0.59 ± 0.11 μm, respectively), yet they resulted in very similar SHP099 loading (∼124 μg and 127 μg, respectively) (Fig. 3b). Furthermore, we found no change in the thickness of (CHT/PβCD)_25_ films before and after incubation in SHP099 solution for “post-assembly” loading with SHP099. When SHP099 is loaded “pre-assembly”, exposed portions of the drug may potentially contribute to charge shielding, reducing electrostatic repulsion between PβCD carboxylic acids, leading to incorporation of larger amounts of polymer into the film during assembly. These interactions may also explain the more rapid film growth observed for (CHT/PβCD–SHP099)_*n*_*versus* (CHT/PβCD)_*n*_. Similar effects have been observed in LbL films assembled under increasing ionic strength (up to a limit).^42,43^ Another possibility is SHP099 guest molecules acting as “crosslinks” if portions of the same guest molecule are incorporated into hosts on two different PβCD chains, providing an additional assembly mechanism, leading to thicker films compared to films without SHP099 or films with SHP099 introduced after film assembly.

**Fig. 4:**
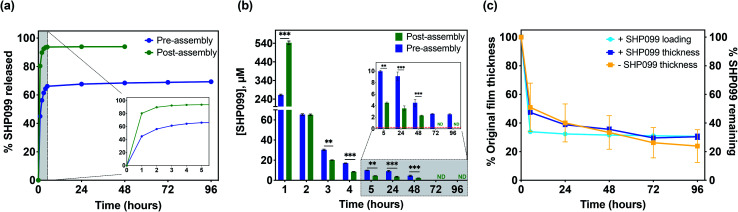
SHP099 release and change in film thickness over time. (a) Normalized cumulative release (percent of total loading) of SHP099 from (CHT/PβCD)_25_ films with SHP099 loaded “pre-assembly” and “post-assembly” incubated in 1× PBS, pH 7.4 at 37 °C. Inset: Release in first 5 hours. (b) Concentration of SHP099 released from “pre-assembly” and “post-assembly” loaded films over time. Inset: Concentration of SHP099 released at 5 hour time point onward. Red dashed line indicates IC_50_ of SHP099 for human esophageal and breast adenocarcinoma cell growth inhibition (0.25 μM). (c) Change in film thickness of (CHT/PβCD)_25_ films loaded with SHP099 (“pre-assembly”) or unloaded over time along with SHP099 release from loaded films. Results are reported as mean ± standard deviation; statistical significance was examined between “pre-assembly” and “post-assembly” loaded films at each time point using Student's *t*-test (unpaired, two-tailed), *n* = 3, **p < 0.01, ***p < 0.001. ND: not detectable.

Although SHP099 loading was similar in both “pre-assembly” and “post-assembly” loaded 25 bilayer films, we were interested in investigating potential differences in drug release *in vitro* that might be caused by drug loading method. These films were incubated in physiologically relevant conditions (1× PBS, pH 7.4 at 37 °C), and film eluent was periodically removed, while refreshing the incubation solution. SHP099 was quantified over time *via* absorbance measurements of these release solutions (Fig. 4a and b). A bolus release of SHP099 was observed over the first 1 to 2 hours of incubation for both “pre-” and “post-assembly” loaded films. For films in which PβCD–SHP099 was complexed “pre-assembly”, these films released 56 ± 1% of their total SHP099 load in this time, while films loaded “post-assembly” demonstrated a more rapid release, leading to 90 ± 1% of their total load being released in this time. After this initial rapid release, films loaded “pre-assembly” continued to release between 2.5 ± 0.1 μM to 9.1 ± 0.7 μM SHP099 daily over 96 hours, at which point SHP099 release fell below our determined experimental detection limit (0.03 μM) (Fig. 4b). Films loaded “post-assembly” continued to release SHP099 up to 48 hours but at lower concentrations than “pre-assembly” loaded films (between 0.05 ± 0.03 μM to 0.5 ± 0.2 μM), after which SHP099 release fell below the detection limit. For both films, at each time point where SHP099 release was detected, this SHP099 release concentration was well above the established half-maximal inhibitory concentration (IC_50_) for human esophageal and breast adenocarcinoma cell growth inhibition (0.25 μM).^1^ Overall, we found that “post-assembly” and “pre-assembly” loaded LbL films released 94 ± 1% and 69 ± 1% of their total load by 48 and 96 hours, respectively (Fig. 4a). The rapid release of nearly all of the SHP099 from “post-assembly” loaded films suggests that loading of SHP099 is likely driven more by absorption and potentially some electrostatic interactions of the SHP099 primary amine and the PβCD carboxylic acids, rather than host–guest interactions of the SHP099 with the PβCD hydrophobic pocket. It is also possible that most of the SHP099 in these films is present within the outer volumes of the film, due to a lack of penetration into the deeper film layers during the loading process. In contrast, we found that although “pre-assembly” loaded films exhibited a large SHP099 release within the first 2 hours, SHP0099 release was sustained over 96 hours and not all of the loaded SHP099 was released in this time. These findings further suggest that PβCD–SHP099 host–guest interactions slow release from these films, following the initial bolus release.

We hypothesize that the initial bolus SHP099 release observed from “pre-assembly” loaded films is likely due to dissolution of the films caused by differences in pH and ionic strength between film assembly conditions (0.1 M sodium acetate buffer, pH 6) and film release conditions (1× PBS, pH 7.4). In order to investigate this hypothesis, we monitored changes in film thickness for (CHT/PβCD–SHP099)_25_ films loaded “pre-assembly” (Fig. 4c) and (CHT/PβCD)_25_ films assembled without any SHP099. Both film architectures exhibited identical dissolution profiles, decreasing in thickness by ∼50% in the first 5 hours of release (accompanied by release of ∼66% of drug for the SHP099 loaded films). Further dissolution of the films over 96 hours proceeded more gradually, with a ∼70% decrease in film thickness overall, regardless of SHP099 loading or not. Dissolution kinetics were similar to SHP099 release kinetics for SHP099 loaded films, indicating initial bolus release is indeed likely caused by film dissolution. After the initial rapid film dissolution over the first 5 hours, film thickness changed gradually, demonstrating that at these later time points, SHP099 is likely released both by diffusion from the film and film dissolution. Additionally, the presence of SHP099 demonstrated no apparent effect on film dissolution rate, suggesting that film destabilization is likely due to differences in incubation solution pH and ionic strength.

### 3.3 Inhibition of cancer cell proliferation *in vitro*

**Fig. 5:**
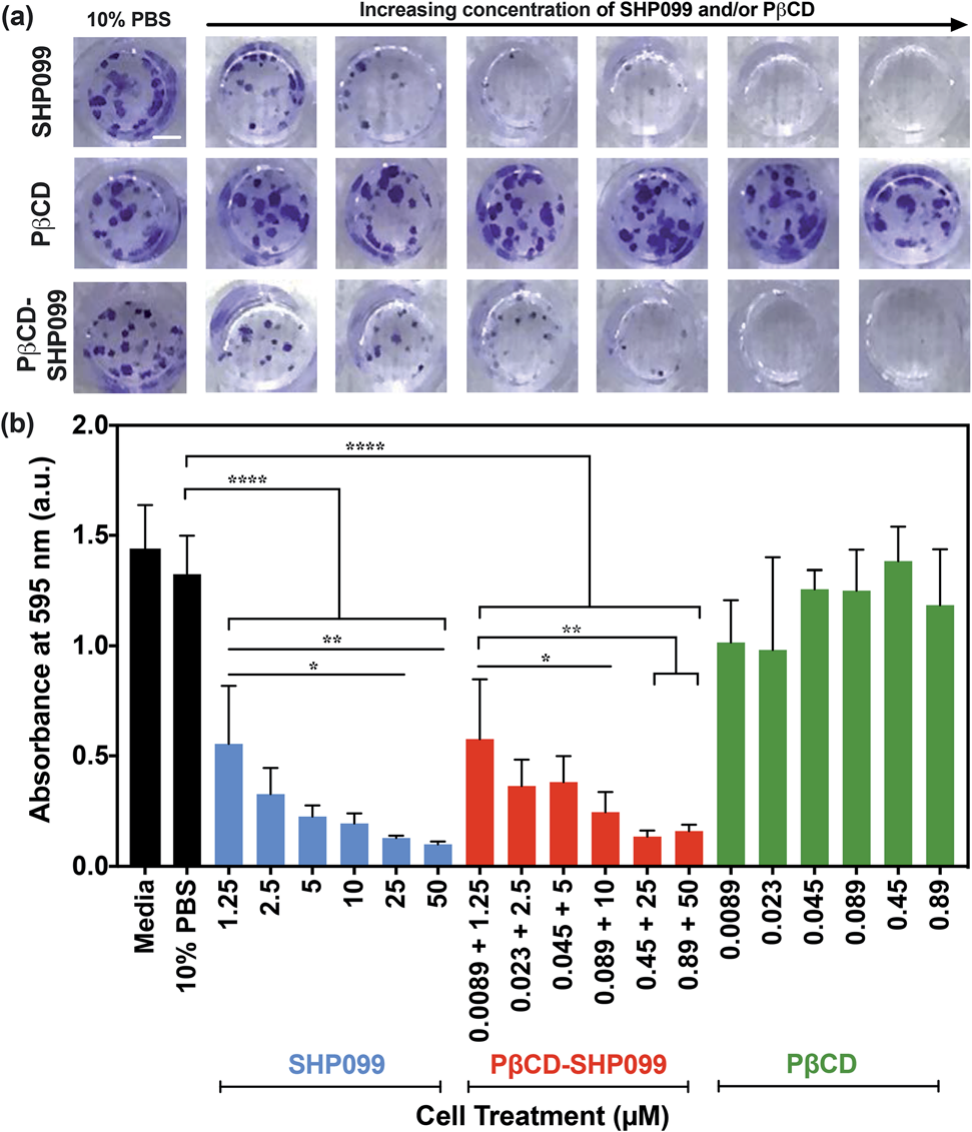
MDA-MB-468 cell proliferation in the presence of SHP099 and PβCD. (a) Representative images of crystal violet stained MDA-MB-468 cell colonies after 14 days of growth in 10% v/v 1× PBS in media, or media containing SHP099 (at increasing concentrations: 1.25, 2.5, 5, 10, 25 and 50 μM), PβCD (at increasing concentrations: 0.0089, 0.023, 0.045, 0.089, 0.45 and 0.89 μM), or PβCD–SHP099 (incubated with both SHP099 and PβCD at the same increasing concentrations) with 10% v/v 1× PBS. Scale bar = 2 mm. (b) Background subtracted absorbance measurements of eluted crystal violet from MDA-MB-468 cells treated with media, 10% v/v 1× PBS in media, SHP099, PβCD–SHP099, or PβCD with 10% v/v 1× PBS in media. Results are reported as means ± standard deviations. Statistical significance was examined using one-way ANOVA and Tukey's post hoc analysis, *n* ≥ 3, *α* = 0.05, *p < 0.05, **p < 0.01, ****p < 0.0001.

The anticancer efficacy of SHP099 LbL films was examined by studying the effect of film release components on MDA-MB-468 colony formation. SHP099 has previously been shown to inhibit the proliferation of MDA-MB-468, a human breast adenocarcinoma cell line.^1^ First, we examined the formation of cancer cell colonies in the presence of non-film-encapsulated SHP099 and PβCD, as well as PβCD–SHP099. Concentrations of SHP099 tested were selected based upon the range of concentrations eluted from (CHT/PβCD–SHP099)_25_ films following initial bolus release, as well as SHP099 concentrations established to be effective against colony formation of the MDA-MB-468 cell line.^1^ For PβCD–SHP099 solutions, these SHP099 concentrations were used with PβCD at a 1 : 2 molar ratio. Finally, the highest and lowest concentrations of PβCD used in these PβCD–SHP099 solutions were used to determine the range of concentrations for PβCD treatment alone. Compared to untreated control cells, the density of cancer cell colonies (stained with crystal violet) was visibly reduced in cells treated with both free SHP099 and pre-mixed PβCD–SHP099 (Fig. 5a). Further, both of these treatment groups exhibited increased inhibition of colony formation with increasing SHP099 concentrations. As expected, we observed no visible difference in colony density for groups treated only with PβCD at any of the tested concentrations compared to untreated controls (Fig. 5a). The crystal violet stain was eluted and quantified *via* absorbance measurements (Fig. 5b). A significant reduction in crystal violet staining was observed for MDA-MB-468 cells exposed to SHP099 compared to untreated controls, with a ∼60% reduction in crystal violet absorbance at even the lowest SHP099 concentration investigated (1.25 μM) (Fig. 5b). In general, inhibition of cell proliferation increased with increasing concentrations of SHP099. This concentration dependence was observed regardless of the presence of PβCD; for each SHP099 concentration, no statistically significant difference was observed in crystal violet absorbance for cells treated with or without SHP099 pre-incubation with PβCD. Visually, there was no difference in crystal violet staining of MDA-MB-468 cells exposed to PβCD alone at all concentrations tested compared with untreated controls. These results confirmed that in cell culture conditions, SHP099 was able to exhibit its anti-proliferative activity against cancer cells. Given that SHP099 exhibits its allosteric inhibition of SHP2 intracellularly,^1^ SHP099 must be able to enter the cell under cell culture conditions. These culture conditions differ from the initial PβCD–SHP099 complexation conditions, and potentially promote release of SHP099 from the PβCD–SHP099 inclusion complex, allowing internalization of the inhibitor.

**Fig. 6:**
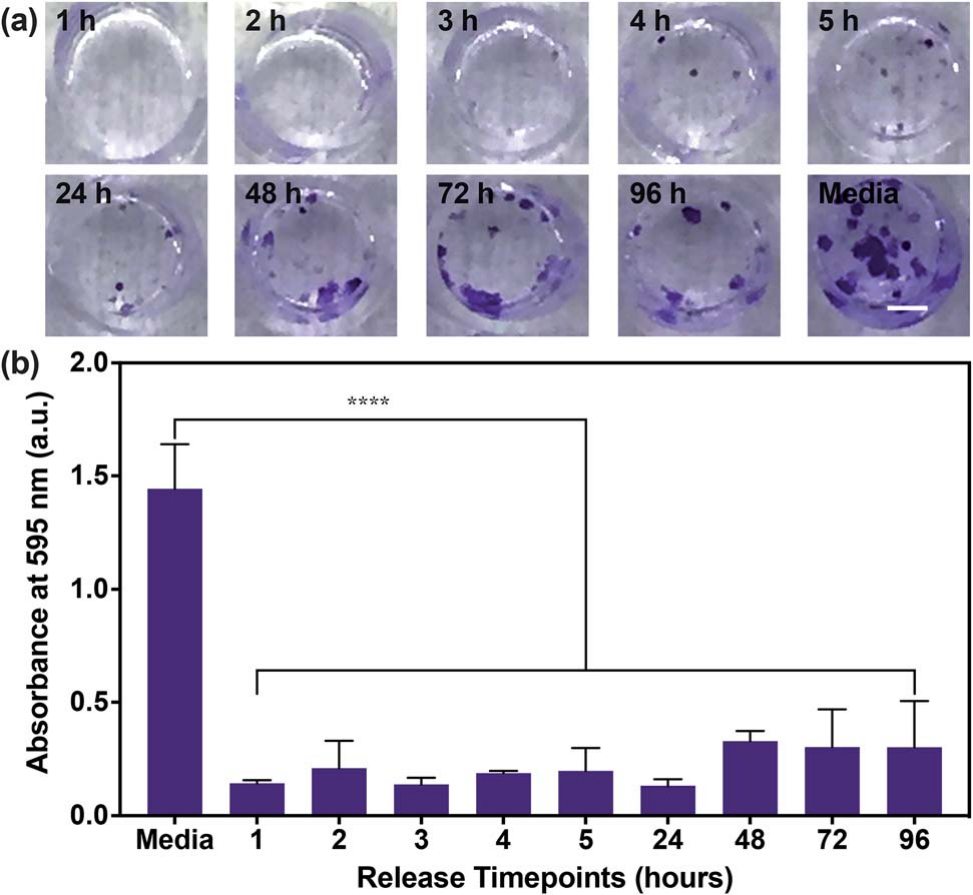
MDA-MB-468 cell proliferation in the presence of (CHT/PβCD–SHP099)_25_ film release media. (a) Representative images of crystal violet stained MDA-MB-468 cell colonies after 14 days of growth in media (untreated cell controls) or media containing film release components from “pre-assembly” loaded (CHT/PβCD–SHP099)_25_ films. Time points at which film release components were collected are indicated on each image. Scale bar = 2 mm. (b) Background subtracted absorbance measurements of eluted crystal violet from MDA-MB-468 cells treated with media or the (CHT/PβCD–SHP099)_25_ release solutions. Results are reported as means ± standard deviations. Statistical significance was examined using one-way ANOVA and Tukey's post hoc analysis compared to untreated controls, *n* ≥ 3, *α* = 0.05, ****p < 0.0001.

Having confirmed the inhibition of MDA-MB-468 proliferation in the presence of SHP099 and PβCD–SHP099, we examined whether the eluent from (CHT/PβCD–SHP099)_25_ LbL films would exhibit this same inhibitory capability for the length of film release. Due to the longer term release of films fabricated with “pre-assembly” loading of SHP099 compared with “post-assembly” loaded films, we used only “pre-assembly” loaded films in these studies. Eluent from different time points (over 96 hours) for films incubated in cell culture media was used to culture MDA-MB-468 cells. Compared to untreated controls, MDA-MB-468 cells cultured in film-incubated media showed significantly reduced colony formation compared to untreated controls, at all time points (Fig. 6a and b). Quantifying crystal violet staining demonstrated that there was no significant difference in colony formation between cells treated with the film release solutions collected at different time points (Fig. 6b), primarily due to variability in colony formation for cells treated with film-incubated media at the later release time points (*e.g.*, 72 hours and 96 hours). Overall, however, all film release samples exhibited a reduction in crystal violet absorbance between ∼80–90% compared to untreated controls. These findings indicate that SHP099 complexation and loading into LbL films does not affect the anticancer activity of this SHP2 inhibitor. Future preclinical studies may aim to investigate growth inhibition of cancer cells in direct contact with these films.

While SHP099 release from (CHT/PβCD–SHP099)_25_ films was able to reduce human breast adenocarcinoma cell growth, it is important to ensure that these films do not exhibit toxicity towards noncancerous cells. Chitosan and cyclodextrin are widely used in biomedical applications due to their well-established biocompatibility.^44,45^ However, SHP099 has only recently begun to be explored as a SHP2 inhibitor, and its cytotoxicity has not been comprehensively investigated to date. As an initial investigation of potential toxicity associated with SHP099, the effect of SHP099 incubation with murine fibroblasts (NIH 3T3) was investigated. These cells were incubated with increasing concentrations of SHP099, over the same range tested with MDA-MB-468 cells (1.25–50 μM) and a cell viability assay was conducted (ESI Fig. S2†). No significant effect on cell viability was seen for SHP099 concentrations below 10 μM. We did observe a drastic decrease in viability at 50 μM SHP099. As SHP099 concentrations above 50 μM were only released at the 1 and 2 hour time points from (CHT/PβCD–SHP099)_25_ films, these films may still be suitable for a localized application. Alternatively, a facile rinse of the coated substrates for 1–2 hours will remove this bolus release, leading to SHP099 concentrations that are still highly effective at inhibiting cancer cell proliferation while maintaining the viability of noncancerous cells.

## 4 Conclusions

Systemic drug delivery is responsible for many of the severe side effects of chemotherapy treatment endured by cancer patients, while simultaneously reducing the maximum potential efficacy of chemotherapeutics. Thus, there is a need for localized delivery over time of these drugs. With this goal, we have assembled polyelectrolyte multilayer films with the biocompatible polymers CHT and PβCD, loaded with the chemotherapeutic SHP099. SHP099 release from our films was above the SHP099 IC_50_ observed to block cell growth pathways in cancer cells,^1^ for at least four days. Film release contents were effective at inhibiting human breast adenocarcinoma cell growth *in vitro* at concentrations below those which were toxic to murine fibroblasts. Our results also indicate the advantage of pre-encapsulation of the drug within PβCD cavities compared to films loaded with SHP099 post-film assembly; the host–guest complexation of SHP099 and PβCD prior to film assembly enables longer release times following an initial bolus release. Our results demonstrate that (CHT/PβCD–SHP099)_25_ films are promising for future clinical applications, including the coating of implants placed at the site of a primary tumor or tumor resection site, to lower chances of tumor recurrence from residual cancerous tissue. These films can improve upon current clinically utilized chemotherapeutic implants by incorporating an emerging, highly effective anticancer therapeutic. Further, the tunable film architecture provides the opportunity to load multiple synergistic drugs due to the versatility of PβCD drug encapsulation, potentially amplifying the inhibitory effect on cancer cell growth.^46^ In summary, in this study we have shown that SHP099 can be effectively incorporated into LbL film-based drug delivery systems for its localized and sustained release. Future work will benefit from *in vivo* studies of this film architecture for a more thorough characterization of biocompatibility, anticancer efficacy, and pharmacokinetics of the incorporated SHP099 providing evidence to further translate this work towards future clinical use.

## Conflicts of interest

There are no conflicts to declare.

## Supplementary Material

RA-010-D0RA03864D-s001
