# Supplementary material for: Controlled delivery of a protein tyrosine phosphatase inhibitor, SHP099, using cyclodextrin-mediated host–guest interactions in polyelectrolyte multilayer films for cancer therapy
Source: RSC Adv. 2020 May 27;10(34):20073–82. doi: 10.1039/d0ra03864d (PMC9054207; doi:10.1039/d0ra03864d)
Supplement: RA-010-D0RA03864D-s001 [file RA-010-D0RA03864D-s001.pdf]

Electronic Supplementary Material (ESI) for RSC Advances.
This journal is © The Royal Society of Chemistry 2020

Supporting Information: Controlled delivery of a protein tyrosine phosphatase inhibitor, SHP099, using cyclodextrin-mediated host-guest interactions in polyelectrolyte multilayer films for cancer therapy

Soobin Wang,[†] Alessia Battigelli,[†,‡] Dahlia Alkekhia,[†,‡] Alexis Fairman,[†,‡] Valentin Antoci,[¶] Wentian Yang,[¶] Douglas Moore,[¶] and Anita Shukla[*,†,‡,§,‖]

†*School of Engineering, Brown University, Providence, RI 02912*

‡*Center for Biomedical Engineering, Brown University, Providence, RI 02912*

¶*Department of Orthopaedics, Warren Alpert Medical School, Brown University and Rhode Island Hospital, Providence, RI 02912*

§*Institute for Molecular and Nanoscale Innovation, Brown University, Providence, RI 02912*

‖*corresponding author*

E-mail: anita_shukla@brown.edu

1 2D NOESY NMR of Controls

(a)

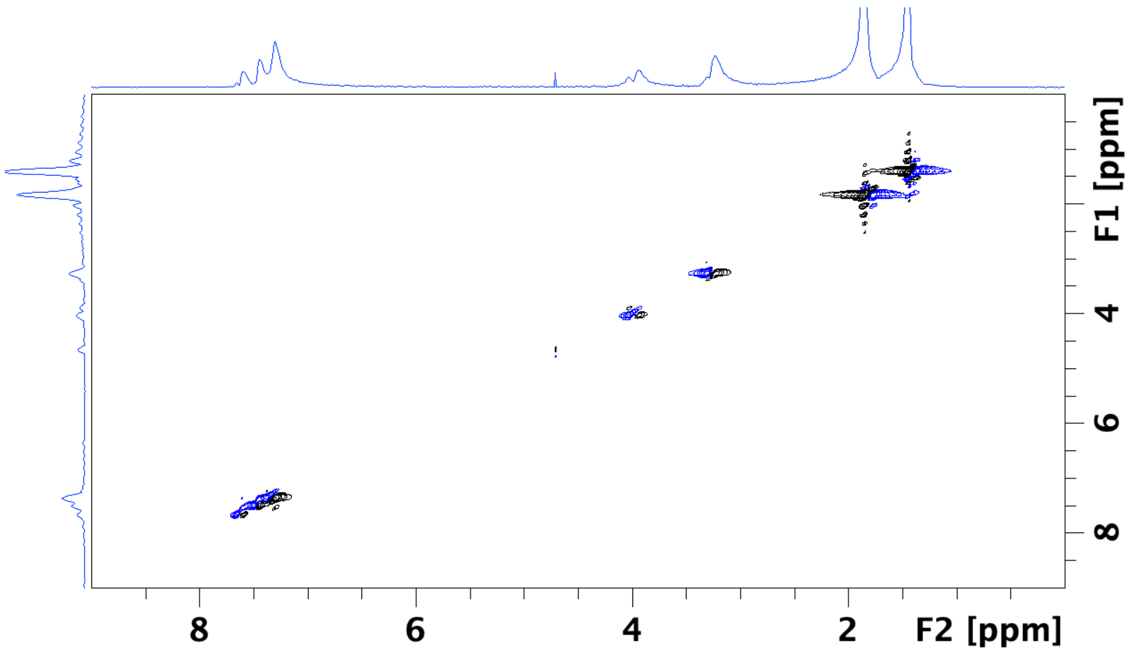

(b)

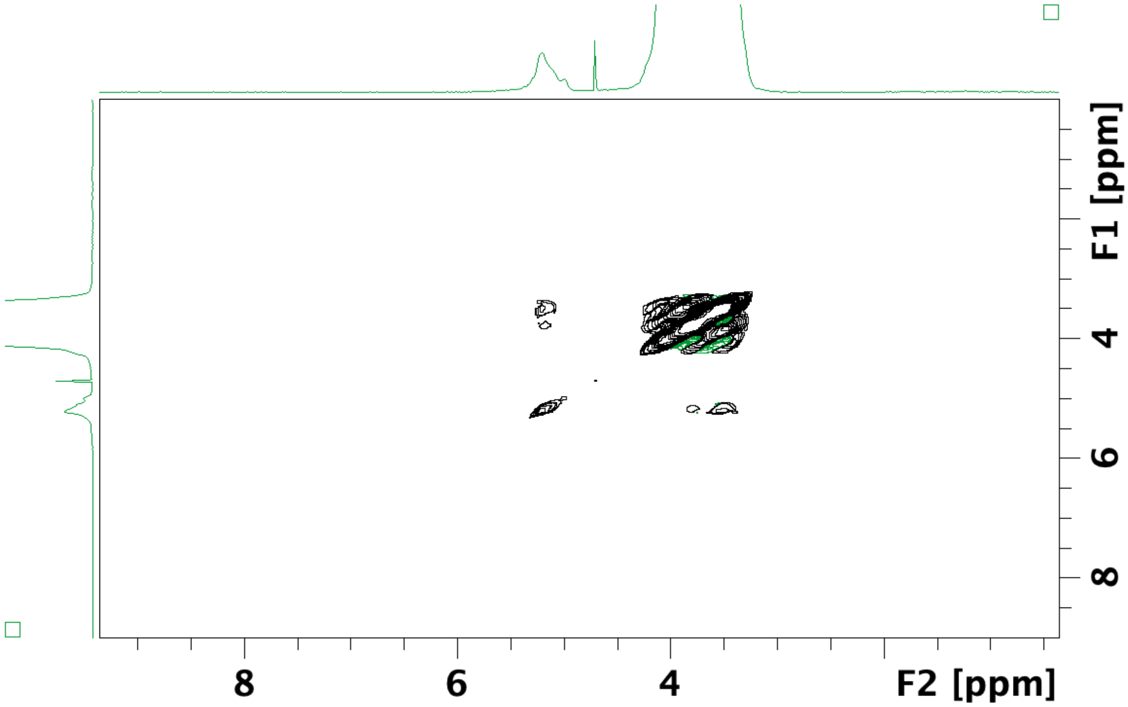

Figure S1: 2D NOESY NMR of (a) SHP099 and (b) PβCD dissolved in D_2O (600 MHz).

2 SHP099 *in vitro* cytocompatibility

Cytocompatibility of SHP099 was assessed for NIH 3T3 murine fibroblasts. NIH 3T3 cells were cultured in DMEM (containing 4 mM l-glutamine, 4500 mg/L glucose, 1 mM sodium pyruvate, and 1500 mg/L sodium bicarbonate) supplemented with 10% v/v calf bovine serum and 1% v/v penicillin–streptomycin at 37°C and 5% CO_2. Cells were seeded in 96-well plates at a seeding density of approximately 2500 cells/cm^2. After 24 h, cells were treated with increasing concentration of SHP099 (from 1.25 to 50 µM), similarly to the clonogenic assays. Cell viability was measured using CCK8 following the vendor protocol. Briefly, after 24 h of SHP099 incubation with the cells, the solutions were removed and 100 µL of CCK8 reagent was added. After 2 h of incubation at 37°C, absorbance at 450 nm was measured using a BioTek® Cytation 3 plate reader. Positive controls of cells grown in DMEM and negative controls lacking cells were included. Normalized cell viability was calculated using Eq. (1).

$$Normalized\ cell\ viability\ \% = \frac{sample\ abs\ -\ negative\ control\ abs}{positive\ control\ abs\ -\ negative\ control\ abs} \times 100 \qquad (1)$$

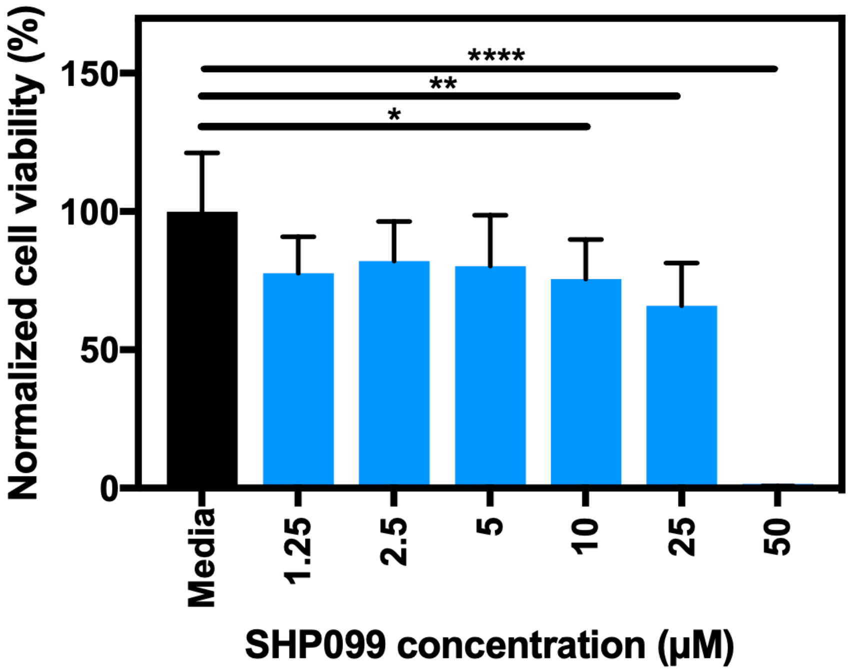

Figure S2: NIH 3T3 fibroblast viability in media incubated with increasing concentration of SHP099 for 14 days to assess biocompatibility of the drug on non cancer cells. Viability and statistical significance are shown relative to the untreated cell control. Results are reported as means ± standard deviations. Statistical significance was examined using one-way ANOVA and Tukey's post-hoc analysis compared to untreated controls, n $\geq$ 3, α=0.05, *p<0.05; **p<0.01; ****p<0.0001; n=3.
